# A no-brainer for ending AIDS: the case for a harm reduction decade

**DOI:** 10.7448/IAS.19.1.21129

**Published:** 2016-04-20

**Authors:** Catherine Cook, Rick Lines, David P Wilson

**Affiliations:** 1 Harm Reduction International, London, United Kingdom; 2 The Burnet Institute, Melbourne, Australia

As Member States gather at the UN General Assembly Special Session (UNGASS) in April 2016, they will discuss strategies for the next 10 years of global drug policy which may inform the high-level meeting on HIV/AIDS in June around declarations for ending the AIDS epidemic by 2030 [1]. At this time, we reflect on how much harm reduction has achieved despite punitive drug policy and legal environments prioritized by most of the world's governments. A recent Lancet Commission on public health and international drug policy implores the world to move away from a war on drugs and to put health at the centre of revolutionizing drug policy [2]. Over the past decade, harm reduction has been proven time and again, across varying countries, regions, social and cultural settings, to be a highly effective HIV prevention measure, a cost-effective set of interventions, and an approach promoting the human rights and dignity of a marginalized and criminalized community. Its adoption in policy and practice has slowly but steadily increased. Today, the majority of the 158 countries with documented injecting drug use have adopted harm reduction measures to some degree in domestic policy and practice: 91 countries allow for harm reduction in national policy documents, 90 have at least one needle-syringe programme (NSP) and 80 countries provide opioid substitution therapy (OST)—an increase of 17 since this monitoring began [3].

Where harm reduction programmes have had adequate financing and the legal and policy space to flourish, the impact has been dramatic. This has been observed among early harm reduction pioneers, as well as in countries to more recently adopt harm reduction. Across Western Europe, there have been low rates of new HIV infections among people who inject drugs (PWID) due to the wide implementation and success of harm reduction policies and services. There and across numerous other settings, regardless of country income status, there is clear evidence that harm reduction implementation is cost-effective [4,5]. As this evidence has mounted, so too has the endorsement of harm reduction from multilateral agencies such as the World Health Organization, UNAIDS and UNODC [6]. By contrast, there have been recent increases in HIV incidence in settings where there have been low and/or reduced access to harm reduction services, such as in Greece, Romania, Pakistan, India, Thailand and the Philippines [7–10].

The clear public health and economic case for harm reduction is further strengthened by steadfast backing from UN human rights mechanisms. Multiple UN human rights bodies now call on governments to implement harm reduction programmes as part of fulfilling the right to the highest attainable standard of physical and mental health, the right to benefit from scientific progress and its applications, and, in places of detention, to freedom from cruel or degrading treatment or punishment. This includes calls for ending compulsory drug detention [11–13].

With this UNGASS comes unequivocal recognition that the global drug policy regime has not achieved the aims previously set [14]. Drug use has not been reduced. Instead, hundreds of billions of US dollars [15] have been misspent on useless and, in many cases, harmful approaches without an evidence or human rights base. Conversely, the potential of harm reduction in many countries has been limited by a lack of strong state support and funding. In many places, services remain small-scale and NGO-driven, not supported to the degree necessary to meet need. While available estimates of global harm reduction investment are outdated, they illustrate a dire situation which will only worsen as donor funds shift away from middle-income countries. At last count, only 7% of the estimated US$2.3 billion required for harm reduction in 2015 was available from international donors [16]. It is clear that funding for these programmes is in crisis. The world has missed the UN target of halving HIV among PWID by 2015 by a staggering 80%, and continuing the status quo will result in a failure to meet the ambitious goal of ending AIDS by 2030.

In October 2015, at the International Harm Reduction Conference, the harm reduction sector released the Kuala Lumpur Declaration [17], calling for alternative responses to drug use that are rooted in evidence, public health, human rights and dignity. The Declaration urges governments and international organizations to adopt harm reduction as a key principle of drug policy throughout the next decade, and to end punitive drug laws, human rights abuses and the mass incarceration of people who use drugs. It also proposes a global target: to redirect 10% of funding from ineffective punitive drug control activities into health and human-rights based programmes, including harm reduction (the “10 by 20” campaign). As the 2016 UNGASS on the World Drug Problem approaches, the many organizations and individuals who have signed this declaration are sending the message that the provision of harm reduction services is no longer a discretional policy option but must be understood as a core obligation of States to meet their international legal obligations under the right to health.

**Figure 1:**
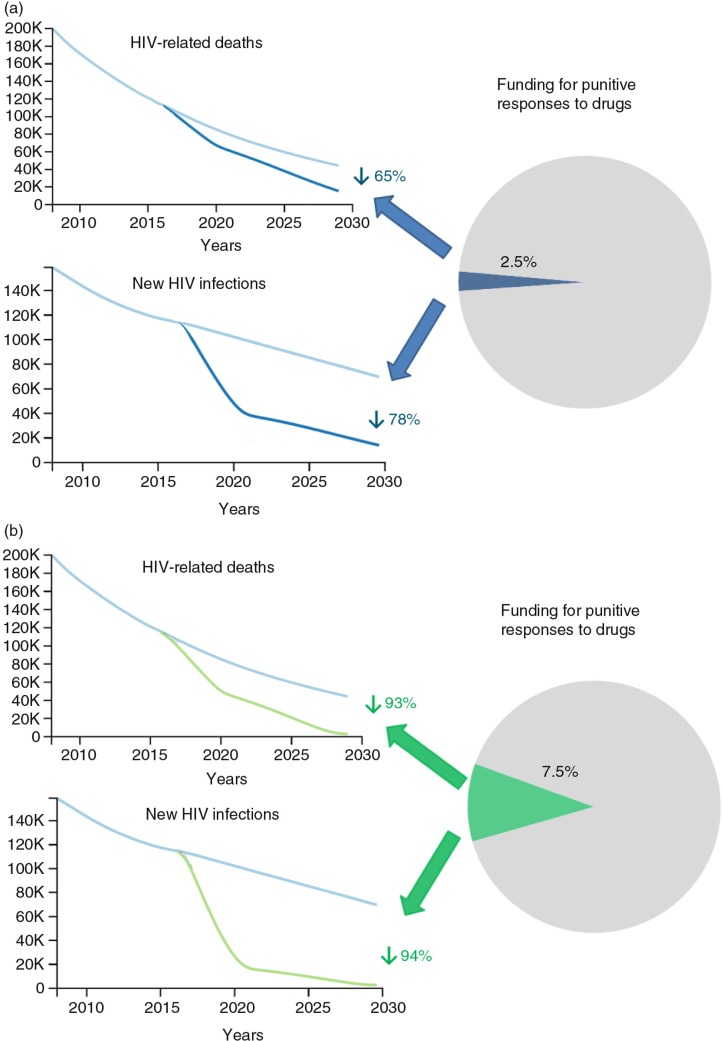
Modelled projections of the global HIV epidemic among PWID comparing the status quo with scenarios where (a) 2.5% of global resources for punitive responses to drugs was used for harm reduction and (b) 7.5% of global resources for punitive responses to drugs was used for harm reduction.

To demonstrate potential impact, we used the Optima model [18] to contrast the minimal impact that continuing the status quo will achieve with the potential of adequately financing harm reduction if policy environments would allow for good access to services. Optima was calibrated to HIV epidemics among PWID in each world region (Asia, Eastern Europe and Central Asia, Western Europe, North America and Oceania, Latin America and the Caribbean, Middle East and North Africa, sub-Saharan Africa) and was then used with region-specific harm reduction cost estimates [4] to produce a global model of HIV among PWID. Future projections were then conducted, revealing that if even a relatively small amount of additional funding were directed into harm reduction, with removal of barriers to service access, the course currently plotted could completely change. A redirection of just 2.5% of the US$100 billion spent each year on drug control [15] could secure a 78% reduction in new HIV infections among PWID by 2030 (Figure 1a). Taking investment to 7.5% of drug control spend has even greater potential, reducing new HIV infections among PWID by a staggering 94% and reducing HIV-related deaths by similar proportions. Thus, the end of AIDS among PWID is thoroughly achievable, contingent on improved policy environments to allow the services to be utilized without harassment or criminalization. What's more, it is achievable through use of a small proportion of the resources currently being used for the target population. This shift towards harm reduction ought to be a no-brainer for governments. The lack of scaled harm reduction is largely due to poor political will that manifests as none or very limited state support. More politically palatable programs are prioritized in many countries despite lower cost-effectiveness ratios. The potential of harm reduction in these settings has not been observed because they have remained small-scale and NGO-driven, not supported to the degree necessary, and are operating despite government and police hostility.

Harm reduction programmes save lives, save money and help respect, protect and fulfil the human rights of people who use drugs. Now is the time to consolidate and secure the success of harm reduction and commit to making the next 10 years The Harm Reduction Decade.
